# Prospective study of fibrosis in the lung endpoints (PROFILE): characteristics of an incident cohort of patients with idiopathic pulmonary fibrosis

**DOI:** 10.1136/bmjresp-2025-003763

**Published:** 2026-01-28

**Authors:** Toby M Maher, Gisli R Jenkins, Gauri Saini, Rebecca Braybrooke, Simon R Johnson, Felix Chua, Pauline T Lukey, Juliet K Simpson, Richard J Allen, Louise V Wain, William A Fahy, Philip L Molyneaux, Iain Stewart

**Affiliations:** 1 USC Keck School of Medicine, Los Angeles, California, USA; 2 Margaret Turner Warwick Centre for Fibrosing Lung Disease, Imperial College London National Heart and Lung Institute, London, England, UK; 3 Respiratory Theme, National Institute for Health Research Imperial Biomedical Research Centre, London, England, UK; 4 Royal Papworth Hospital, Cambridge, England, UK; 5 Respiratory Medicine, Clinical Sciences Building, Nottingham City Hospital, Hucknall Road, University of Nottingham, Nottingham, England, UK; 6 Centre for Respiratory Research and NIHR Biomedical Research Centre, School of Medicine, University of Nottingham, Nottingham, England, UK; 7 Royal Brompton Hospital, London, London, UK; 8 Target to Treatment Consulting Ltd, Stevenage Bioscience Catalyst, Stevenage, UK; 9 GlaxoSmithKline Research & Development Limited, London, England, UK; 10 NIHR Leicester Biomedical Research Centre, Leicester, England, UK; 11 Genetic Epidemiology Group, Department of Health Sciences, University of Leicester, Leicester, UK

**Keywords:** Idiopathic Pulmonary Fibrosis

## Abstract

**Background:**

Idiopathic pulmonary fibrosis (IPF) is a chronic, progressive fibrotic lung disease. Prospective study of fibrosis in the lung endpoints (PROFILE) was a prospective, observational cohort study designed to better define the natural history of IPF, understand disease biology and identify biomarkers to support disease management and enhance clinical trial design.

**Methods:**

Individuals with an incident diagnosis of IPF were recruited between 2010 and 2017 across two co-ordinating centres in the UK. Demographics, clinical measurements and blood samples were obtained at baseline, and 1, 3, 6, 12, 24 and 36 months. Disease progression events were defined as death or relative forced vital capacity (FVC) decline >10% at 12 months. Survival estimates were modelled using Cox proportional hazards; longitudinal lung function decline was estimated using mixed effect models, specified with restricted cubic splines, a random intercept for participant and random effect for study visit. All models were adjusted for baseline age, sex and continuous baseline percent predicted FVC (ppFVC).

**Results:**

A total of 632 participants were recruited, 77.1% were male, and mean age at enrolment was 70.4 years (SD 8.4). Mean baseline ppFVC was 79.5% (SD 19.2), and mean percent predicted DL_CO_ (ppDL_CO_) was 45.7% (SD 15.1). A total of 304 (48.1%) participants met disease progression criteria at 1 year. Median survival was 3.7 years (95%CI 3.3 to 4.0). More severe baseline physiology, 12-month relative lung function decline ≥10%, older age and short telomeres were independent risk factors for mortality. Twelve-month estimated change in ppFVC was −5.28% (95% CI −6.34 to −4.22) with an average FVC decline of 186.9 mL (95% CI −225.4 to −148.5); 12-month estimated change in ppDL_CO_ was −3.35% (95% CI −4.30 to −2.40).

**Conclusion:**

The PROFILE cohort confirms that untreated IPF is inexorably progressive and inevitably fatal with a poor median survival from diagnosis.

**WHAT IS ALREADY KNOWN ON THIS TOPIC**

- Idiopathic pulmonary fibrosis is a progressive disease with variable course yet ultimately fatal. The PROFILE study was a prospective longitudinal study of patients with newly diagnosed IPF to establish insights into the natural history of disease and molecular profiling.

**WHAT THIS STUDY ADDS**

- The PROFILE cohort is a valuable resource that supports consistent interpretation of natural history of IPF. This study presents the protocol including the baseline characteristics and outcomes for the final study cohort.

**HOW THIS STUDY MIGHT AFFECT RESEARCH, PRACTICE OR POLICY**

- This study supports transparency in study design and outcomes, facilitating collaborative efforts that generate insights into the molecular profile of IPF.

## Introduction

 Idiopathic pulmonary fibrosis (IPF) is a chronic, progressive, fibrosing interstitial lung disease (ILD) characterised by a radiologic and/or histopathologic pattern of usual interstitial pneumonia (UIP).^1^ The course of IPF is variable but is ultimately fatal. Systematic review and meta-analysis of international mortality rates has estimated overall 5-year cumulative survival at 45.6% (95% CI 41.5 to 49.7);^2^ median survival was estimated between 2 and 4 years in studies prior to anti-fibrotic therapy.^3^

The clinical tools currently used to assess IPF all have important limitations when it comes to making diagnoses, deciding best management, assessing treatment outcomes for individual patients and as endpoints in clinical trials.^4 5^ Pharmacogenomic studies have suggested that common IPF variants may distinguish treatment response,^6^,^8^ underpinning novel pharmacogenetic trials.^9^ To improve clinical management, it is important to understand the natural history of IPF for which molecular profiling can elucidate the heterogeneity in disease course and inform future precision medicine strategies.

Prospective study of fibrosis in the lung endpoints (PROFILE) was a prospective longitudinal study of patients with newly diagnosed IPF with the following aims: (1) prospective validation of previously published IPF biomarkers, (2) discovery of novel candidate biomarkers to enable disease stratification and for use as surrogate endpoints in future interventional trials and (3) evaluation of longitudinal IPF behaviour to identify potential disease endotypes and to inform the development of clinical endpoints for future interventional studies. The PROFILE study has contributed to advancements in disease stratification and evaluation of longitudinal behaviour (see online supplemental appendix 1 for peer-reviewed publications); however, a diagnosis of IPF continues to be associated with significant mortality risk and limited treatment options in a landscape of rising global ILD burden.^10^

Here, we describe the PROFILE study protocol including the baseline characteristics and outcomes for the final study cohort.

## Methods

PROFILE is a prospective, non-interventional, longitudinal study conducted at multiple centres, coordinated by two recruitment hubs: The Royal Brompton & Harefield NHS Trust, and Nottingham University Hospitals NHS Trust. Ethical approval for the PROFILE study was granted by the Royal Free Hospital Research Ethics Committee (REC 10/H0720/12) and PROFILE (Central England) Northampton Research Ethics Committee (REC 10/H0402/2). All patients provided written, informed consent before participating in the study.

Recruitment into the PROFILE study occurred between September 2010 and September 2017. Subjects were followed until death or June 2020. Subjects were eligible for inclusion if they were >18 years of age and had a multidisciplinary team diagnosis of IPF according to international guidelines^11^ within 6 months of enrolment. Subjects were excluded if they had co-existent conditions known to be associated with the development of fibrotic lung disease (eg, connective tissue disease, suspected drug-induced lung disease, asbestosis or granulomatous disease including sarcoidosis). In addition, subjects with co-morbid disease severity that in the opinion of the investigators gave them an expected life expectancy of less than 1 year were excluded, as were those participating in clinical trials assessing novel IPF therapies and those unable to provide consent.

Following the provision of signed informed consent, subjects completed a baseline visit. This included clinical assessment, measurement of lung function, 6-min walk test (6MWT) and patient-reported outcome measures (St Georges Respiratory Questionnaire (SGRQ), Leicester Cough Questionnaire (LCQ) and mMRC dyspnoea score). If clinically indicated, subjects underwent thoracic CT. Blood samples were drawn for serum and plasma, PAXgene (RNA) and whole blood for DNA analysis. Subjects who underwent clinically indicated bronchoscopy also provided consent for surplus bronchoalveolar lavage to be kept for research purposes. Subjects were followed up at 1, 3, 6, 12, 24 and 36 months (online supplemental table 1 and table 2.

Percentages are reported for categorical data. Mean and SD are presented for continuous normally distributed data. Medians and IQRs are presented for non-normal data. Percent predicted FVC (ppFVC) and ppDL_CO_ are derived using GLI.^12^ Composite physiological index (CPI) was calculated as previously described.^13^ Gender-Age-Physiology (GAP) index was derived using sex, age and lung function as previously described.^14^ WHO Nutritional Status was used to classify BMI values.^15^ Single nucleotide polymorphisms at genome-wide significance were genotyped, and the frequency of the effector allele was calculated. Computational telomere length was generated using Telseq v0.0.2 with a repeat number of 10,^16^ subsequently used to derive telomere length quintiles.

Disease progression events were defined as death or relative FVC decline >10% at 12 months. Case-by-case adjudication of disease progression was performed by clinical teams where 12-month lung function data were missing. A mixed effects model, adjusted for baseline age, sex and baseline ppFVC, specified with restricted cubic splines using three knots, a random intercept for participant and random effect for study visit, was used to estimate the mean change in lung function between baseline and 12 months. Median follow-up is presented with IQR, median survival is presented with 95% CIs. Cumulative crude mortality was described at 1, 3 and 5 years. Cox proportional hazards models were specified to estimate the risk of mortality events during follow-up according to demographic and clinical characteristics, adjusted for age, sex, baseline ppFVC and reported as unadjusted or adjusted hazard ratio (aHR). Estimates for associations with *MUC5B* genotype were based on an additive model restricted to participants with genetically inferred European ancestry and further adjusted for the first three genetic principal components, derived from genome wide array genotyping. For genetically inferred ancestry, data were merged with HapMap samples as a global population reference panel and principal components analysis were performed.^17^ K-means clustering was performed on the first two genetic principal components, optimised to distinguish a cluster of individuals identifying as European. Follow-up time was censored at 5 years to meet proportional hazard assumptions, checked using Schoenfeld residuals. To estimate the mortality risk corresponding to 12-month estimated lung function decline, participants were considered at risk of mortality from 12 months with events before this timepoint excluded. Sensitivity analysis on mortality risk was performed in individuals with no evidence of anti-fibrotic use or steroid use at baseline or during follow-up. Stata 18.0 was used for all statistical analyses.

## Results

A total of 632 participants were recruited to the PROFILE study (table 1). Mean age in the cohort was 70.4 years (SD 8.4 years). A total of 302 participants were recruited from the Brompton (47.8%) and 330 from the Nottingham co-ordinating centre (52.2%). The cohort was predominantly male (n=487; 77.1%) and self-reported as white (n=608; 96.2%). Ex-smokers comprised 398 (63.0%) participants, 201 (31.8%) were never smokers and 32 (5.1%) were current smokers. The WHO Nutritional Status criteria for pre-obesity were met in 45.1% (n=285) of the cohort.

**Table 1:** Baseline demographics and disease severity (n=632)

Sex: n %		
Male	487	77.1%
Female	145	22.9%
Ethnicity: n%		
White	608	96.2%
Non-White	24	3.8%
WHO nutritional classification: n %		
Underweight	7	1.1%
Normal	124	19.6%
Pre-obese	285	45.1%
Obese classes I–III	211	33.4%
Body mass index (BMI)		
BMI: mean (SD)	28.3	(4.3)
Smoking status: n %		
Never	201	31.8%
Ex-smoker	398	63.0%
Current	32	5.1%
Age category: n %		
Under 50	8	1.3%
50–59	60	9.5%
60–69	223	35.3%
70–79	262	41.5%
80 and over	79	12.5%
Age: mean (SD)		
Years	70.4	(8.4)
Lung function parameters: mean SD		
FVC (L)	2.76	0.85
ppFVC (%)	79.54	19.17
DL_CO_	3.78	1.39
ppDL_CO_ (%)	45.72	15.12
ppFEV1/FVC (%)	81.09	7.52
CPI	46.74	13.05
Gender-Age-Physiology (GAP) index: n (%)		
GAP stage I	223	35.3%
GAP stage II	272	43.0%
GAP stage III	118	18.7%
6-min walk test (n=381)		
Metres: mean (SD)	358.5	(102.2)
Desaturation: n%	97	25.5%
mMRC dyspnoea scale: n %		
1	82	13.0%
2	213	33.7%
3	142	22.5%
4	68	10.8%
5	40	6.3%
Patient reported outcome		
SGRQ overall: mean (SD)	45.4	(20.4)
LCQ overall: median (IQR)	16.1	(12.5–18.9)

CPI, composite physiological index; FVC, forced vital capacity; LCQ, Leicester Cough Questionnaire; mMRC, modified Medical Research Council; ppDL_CO_, percent predicted diffusion capacity of carbon monoxide; ppFVC, percent predicted FVC; SGRQ, St Georges Respiratory Questionnaire.

Comorbidity and concomitant medication information was reported in 580 participants (91.8%), for whom the most frequently reported comorbidity was hypertension in 287 participants (49.5%) (online supplemental figure 1). Coronary heart disease and type II diabetes were reported in 104 (17.9%) and 99 (17.1%) participants, respectively. Depression or anxiety was reported as a comorbidity in 67 participants (11.6%), and 36 participants had cancer or a history of cancer at baseline assessment (6.2%). Proton-pump inhibitors were recorded as concomitant medication in 307 participants (52.9%) (online supplemental figure 2), 148 participants had a record of mucolytics (25.5%) and 71 participants had a record of steroid or immunosuppression (12.2%). A total of 55 participants had a record of nintedanib or pirfenidone at baseline (9.5%); a further 114/632 (18.0%) received an antifibrotic drug during study follow-up.

Mean ppFVC at baseline was 79.5% (SD 19.2) and ppDL_CO_ of 45.7% (SD 15.1) (table 1). The cohort had a mean CPI of 46.7 (SD 13.1). Most participants had mild to moderate disease according to GAP staging; 223 (35.3%) subjects were GAP stage 1 and 272 (43.0%) GAP stage II and 118 (18.7%) GAP stage III. Information on 6MWT was available for 381 participants (60.3%), who had a mean walk distance of 358.5 m (SD 102.2 m) with 25.5% reaching the 88% desaturation threshold. Most participants 213 (33.7%) reported breathlessness on the MRC scale at grade 2 (n=213, 33.7%) and grade 3 (n=142, 22.5%). The mean SGRQ score was 45.4 (SD 20.4), and the median LCQ score was 16.1 (IQR 12.5–18.9). Genotype information was available for 534 (84.5%) participants, including a minor allele frequency of *MUC5B* rs35705950 risk allele of 34.2% in the overall cohort (online supplemental table 3).

Disease progression at 12 months was recorded in a total of 304 participants (48.1%) (table 2). Twelve-month estimated change in ppFVC was −5.28% (95% CI −6.34 to −4.22). Twelve-month estimated change in FVC was −186.9 mL (95% CI −225.4 to −148.5). Twelve-month estimated change in ppDL_CO_ was −3.35% (95% CI −4.30 to −2.40). Overall median survival was 3.7 years (95% CI 3.3 to 4.0) (table 2, online supplemental figure 3). The cumulative mortality rate was 16.1%, 42.6% and 60.4% for 1 year, 3 years and 5 years, respectively. Participants recorded as GAP stage I at baseline had a median survival of 6.7 years (95% CI 6.1 to 7.6), GAP stage II median survival was 3.2 years (95% CI 2.6 to 3.6) and GAP stage III median survival was 1.8 years (95% CI 1.3 to 2.1).

**Table 2:** Overview of clinical outcomes (n=632)

Disease progression		N	%
	Progressor	304	48.1%
	Not progressor	328	51.9%
12-month lung function change		Mean	95% CI
	Estimated FVC (ml)	−186.9	−225.4 to −148.5
	Estimated ppFVC (%)	−5.28	−6.34 to −4.22
	Estimated ppDL_CO_ (%)	−3.35	−4.30 to −2.40
Mortality rate (crude)		n	%
	1 year	102	16.1%
	3 years	269	42.6%
	5 years	382	60.4%
Median survival (years)		median	95%CI
	Overall	3.7	3.3 to 4.0
	GAP stage I	6.7	6.1 to 7.6
	GAP stage II	3.2	2.6 to 3.6
	GAP stage III	1.8	1.3 to 2.1

Disease progression events were defined as death or relative FVC decline >10% at 12 months. Case-by-case adjudication of disease progression was performed by clinical teams where 12-month lung function data were missing. Estimated 12-month change in lung function modelled with mixed effects, adjusted for baseline age, sex and ppFVC, specified with restricted cubic splines using three knots, a random intercept for participant and random effect for study visit.

FVC, forced vital capacity; GAP, Gender-Age-Physiology Index; ppDL_CO_, percent predicted diffusion capacity of carbon monoxide; ppFVC, percent predicted FVC.

**Figure 1:**
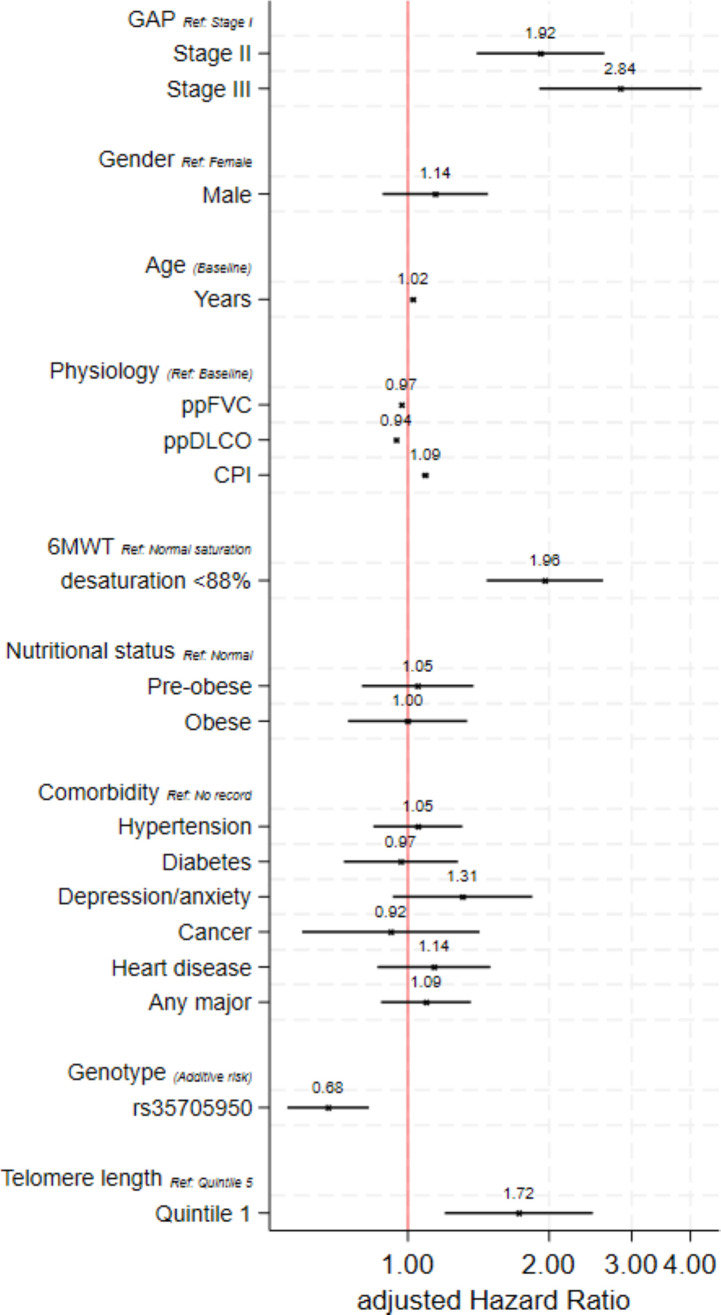
Adjusted risk of mortality according to baseline characteristics. Estimates modelled using cox proportional hazards adjusted for continuous age at baseline, sex and continuous baseline percent predicted forced vital capacity (ppFVC). Follow-up time censored at 5 years. *MUC5B* rs35705950 was restricted to European ancestry, further adjusted for first three genetic principal components, with risk allele included in an additive model. Telomere length presented as quintiles (**Q**) with Q5 representing greatest length and Q1 representing shortest length. Gender-Age-Physiology index (GAP); percent predicted carbon monoxide diffusion capacity (ppDL_CO_); 6-min walk test (6MWT), SpO2 desaturation <88%.

Over the duration of follow-up, relative to GAP stage I, participants recorded as GAP stage II at baseline were at a 91% greater risk of mortality (aHR 1.91, 95% CI 1.40 to 2.62, p <0.001), and GAP stage III were at over twofold greater risk of mortality (aHR 2.84, 95% CI 1.91 to 4.22, p<0.001) (figure 1, online supplemental table 4). Male sex was not independently associated with mortality (aHR 1.14, 95% CI 0.88 to 1.48, p=0.31), a 2% greater risk of mortality was associated with incremental age in years (aHR 1.02, 95% CI 1.01 to 1.04, p<0.001), a 3% lower risk of mortality was associated with each incremental unit change in ppFVC (aHR 0.97, 95% CI 0.96 to 0.98, p<0.001) and a 6% lower risk of mortality was associated with each incremental unit change in ppDL_CO_ (aHR 0.94, 95% CI 0.94 to 0.95, p<0.001). Incremental CPI was associated with 9% greater risk of mortality (aHR 1.09, 95% CI 1.07 to 1.11, p<0.001). Desaturation <88% post 6MWT was associated with greater risk of mortality (HR 1.96, 95% CI 1.47 to 2.61, p<0.001).

WHO Nutritional Status of pre-obese or obesity (classes I–III) was not significantly associated with risk of mortality compared with normal status (aHR 1.05, 95% CI 0.80 to 1.38, p=0.731 and aHR 1.00, 95% CI 0.75 to 1.34, p=0.991 respectively) (figure 1, online supplemental table 4). In adjusted analyses, neither hypertension, diabetes, depression/anxiety, cancer, heart disease, nor any record of these major comorbidities combined was significantly associated with mortality. The *MUC5B* rs35705950 risk allele was associated with a 32% lower risk of mortality in participants with European ancestry (aHR 0.68, 95% CI 0.55 to 0.83, p<0.001). No association was observed on lung function change (online supplemental figure 4). Relative to participants within the greatest quintile of telomere length, those in the lowest quintile were at greater risk of mortality (aHR 1.72, 95% CI 1.20 to 2.48, p=0.003), no significant associations were observed according to common risk variants for *TERC* rs9811216, *TERT* rs2736100 or *RTEL1* rs115610405 (online supplemental table 4). Findings were consistent in sensitivity analysis restricted to 400 participants with no evidence of anti-fibrotic use or steroid use (online supplemental figure 5).

**Figure 2:**
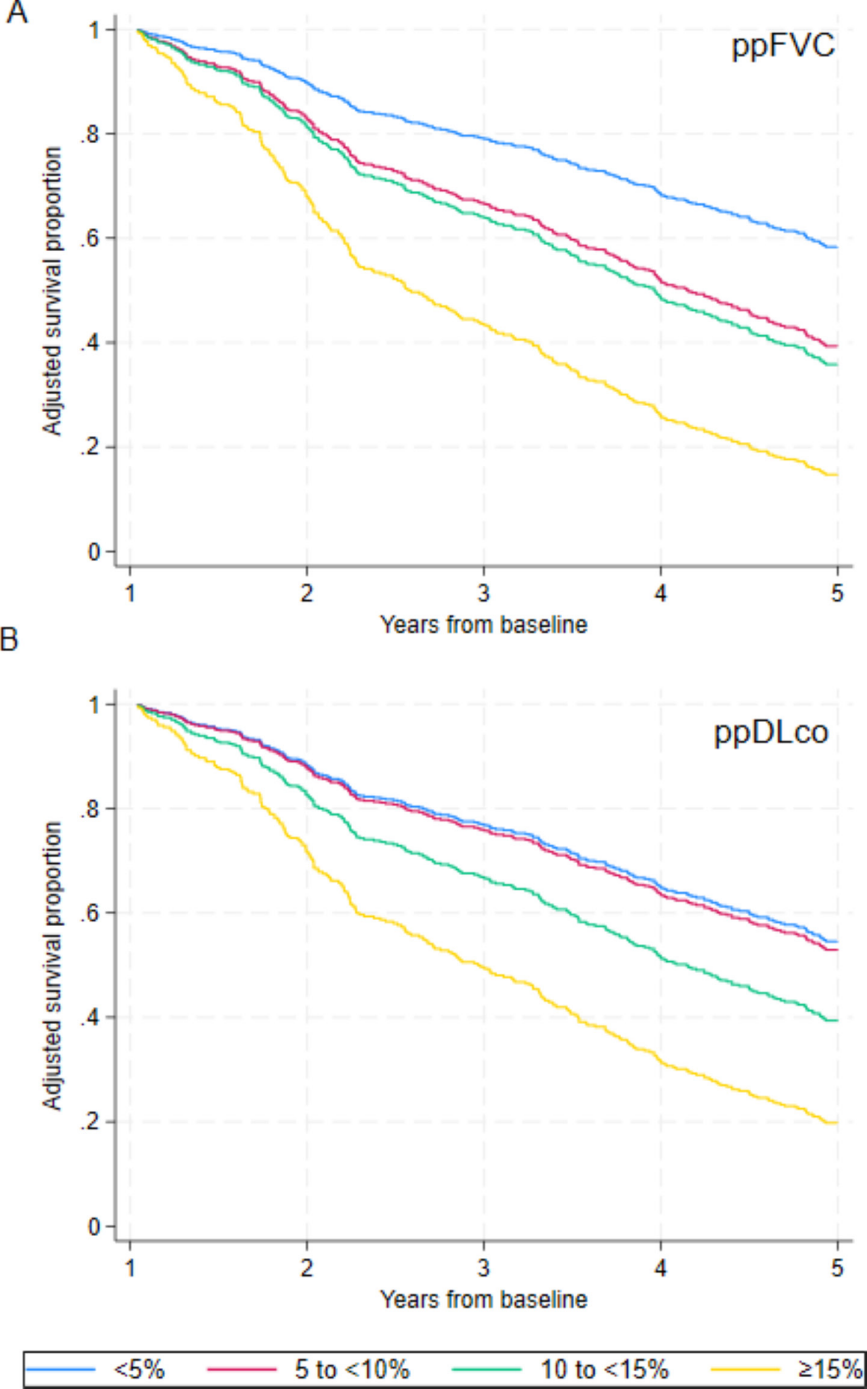
Adjusted survival according to 12-month relative lung function decline. Adjusted survival curves for 12-month relative decline categories of (A) percent predicted forced vital capacity (ppFVC); (**B**) percent predicted carbon monoxide diffusion capacity (ppDL_CO_); based on <5% (blue), 5% to <10% (red), 10% to <15% (green) and ≥15% (yellow) categories estimated using mixed effect models, adjusted for baseline age, sex and ppFVC, specified with restricted cubic splines using three knots, a random intercept for participant and random effect for study visit.

Based on estimated lung function change over 12 months from baseline, a relative FVC decline of 5%, 10% and 15% was associated with aHR 1.73 (95% CI 1.27 to 2.36, p<0.001), aHR 1.86 (95% CI 1.30 to 2.67, p<0.001) and aHR 3.54 (95% CI 2.52 to 4.99, p<0.001), respectively, when compared with participants who had a decline of <5% (figure 2A, online supplemental table 4). Findings were largely consistent in sensitivity analysis, CIs were wider, and significant association was observed with 15% relative decline (aHR 3.45, 95% CI 2.20 to 5.38, p<0.001) (online supplemental figure 6A). A relative ppDL_CO_ decline of 5%, 10% and 15% was associated with aHR 1.03 (95% CI 0.72 to 1.48, p=0.86), aHR 1.54 (95% CI 1.06 to 2.24, p=0.025) and aHR 2.67 (95% CI 1.87 to 3.82, p<0.001), respectively, when compared with participants who had a decline <5% (figure 2B (online supplemental table 4). Significant findings were consistent in sensitivity analysis (online supplemental figure 6B).

## Discussion

PROFILE is a large prospective IPF cohort study that combines longitudinal clinical follow-up with biological sample collection. Recruitment into PROFILE was initiated prior to the approval of antifibrotic therapy and therefore provides an understanding of the untreated natural history of IPF. Other unique features of the PROFILE study include the requirement that patients have an incident IPF diagnosis at recruitment, the use of standard operating procedures across sites, longitudinal sample collection with matching clinical information and the availability of long-term follow-up.

The clinical characteristics of the PROFILE are consistent with those of previous retrospective studies and other subsequent IPF cohort studies and registries.^18^,^20^ While two antifibrotic therapies have been approved in the UK for IPF (pirfenidone in 2012 and nintedanib in 2014),^21^ the PROFILE cohort was largely anti-fibrotic naïve at enrolment and during follow-up. This makes the cohort especially valuable when it comes to understanding the natural history and pathogenesis of IPF. At baseline, the PROFILE cohort had moderate physiological impairment as measured by FVC and DL_CO_. Almost half of the cohort met the threshold for disease progression in 1 year with an overall median survival of 3.7 years. Adjusted analyses demonstrate that while the GAP score was associated with survival, the effect of sex on mortality was not independent from the effect of age and lung function severity. Lung function change over time is an important prognostic indicator, routinely used in clinical management and increasingly used as clinical trial inclusion criteria for non-IPF fibrotic ILD.^22^ The availability of longitudinal lung function in PROFILE confirms the importance of both FVC and DL_CO_ decline as predictors of future poor prognosis.

The reliable clinical classification of IPF in the PROFILE cohort has contributed to discovery and validation of genetic variants associated with disease susceptibility and progression. *MUC5B* rs35705950 is the common variant with the greatest reported penetrance, with a minor allele frequency of 38% in IPF population studies and 34% reported in PROFILE.^23^ Consistently with large cohort studies of European ancestry suggesting a 22% lower risk of mortality,^24^ the IPF diagnosis risk allele rs35705950 had a protective effect on prognosis as each copy was associated with a 32% lower risk of mortality in the PROFILE cohort. No significant associations were observed between the *MUC5B* risk allele and longitudinal lung function, consistent with findings as part of a larger international analysis.^25^ Telomere length has been reported to be causal for IPF diagnosis and demonstrated to lead to distinct outcomes in pharmacogenetic study.^26 27^ Relative to PROFILE participants with the longest telomeres, participants with the shortest telomeres were associated with a 72% greater risk of mortality. No associations with mortality were observed for common IPF susceptibility risk variants in telomere-related genes, rare telomere-related variants have been robustly implicated in IPF prognosis.^28^

A major strength of the PROFILE study is in its prospective design to comprehensively investigate the natural history of disease. To the best of our knowledge, it represents the largest incident untreated disease cohort of IPF. A small subgroup was originally diagnosed with idiopathic non-specific interstitial pneumonia with no major mechanistic or biological distinctions.^29^ On re-evaluation under the latest international guidelines, these participants meet the criteria for indeterminate IPF.^30^ The prospective goal to study disease mechanisms informed the protocolised, longitudinal bio-banking of sera and plasma. This has subsequently enabled genetic studies through whole genome sequencing and microarray platforms,^31 32^ RNA sequencing^33^ and affinity-based and spectrometry-based proteomics.^34^,^37^ The availability of routine radiological imaging has permitted the use of radiomics,^38^ and groups of patients from the PROFILE cohort have been studied using home handheld spirometry or bronchoscopy that enabled assessment of the lung microbiome.^39 40^ The availability of these technologies combined with careful clinical phenotyping and longitudinal assessment has contributed to the development of novel clinical endpoints and advanced trial design.^41 42^

An anticipated limitation with the focused recruitment of incident IPF in a UK multicentre study is the restricted diversity with regards to ethnicity, non-IPF disease, age and sex, which may affect generalisability. Recruitment and protocolised follow-up were performed during an observation period prior to licensing of antifibrotics for usual care, which limits the interpretation of the effects of antifibrotic therapy on lung function decline and mortality, as well as the effect of treatment on biomarkers. Thus far, analysis of the cohort has only achieved a fraction of its potential. The study steering committee welcomes data access requests that can leverage novel insights into the natural history of IPF to improve clinical management and create meaningful patient benefit.

The PROFILE study is a prospective, longitudinal multicentre cohort of patients with an incident diagnosis of IPF based on international criteria. Longitudinal sample collection, biobanking and deep-phenotyping data have contributed to a better understanding of IPF disease behaviour and biology.

### Patient and public involvement

A patient and public involvement representative from charity organisation, Action for Pulmonary Fibrosis, attended each PROFILE steering committee meeting. Participant representatives attended a Midlands Interstitial Lung Disease Research Day to support dissemination of study findings.

## Supplementary material

10.1136/bmjresp-2025-003763 online supplemental file 1

10.1136/bmjresp-2025-003763 online supplemental file 2

## Data Availability

Data are available upon reasonable request.
