# Supplementary material for: Prospective study of fibrosis in the lung endpoints (PROFILE): characteristics of an incident cohort of patients with idiopathic pulmonary fibrosis
Source: BMJ Open Respir Res. 2026 Jan 28;13(1):e003763. doi: 10.1136/bmjresp-2025-003763 (PMC12853555; doi:10.1136/bmjresp-2025-003763)
Supplement: online supplemental file 1 [file bmjresp-13-1-s001.docx]

| **Year** | **Title** | **Journal** | **First author** | **DOI** |
| --- | --- | --- | --- | --- |
| 2015 | Longitudinal change in collagen degradation biomarkers in idiopathic pulmonary fibrosis: an analysis from the prospective, multicentre PROFILE study | Lancet RM | Jenkins | https://doi.org/10.1016/s2213-2600(15)00048-x |
| 2016 | Daily Home Spirometry: An Effective Tool for Detecting Progression in Idiopathic Pulmonary Fibrosis | AJRCCM | Russell | https://doi.org/10.1164%2Frccm.201511-2152OC |
| 2017 | An epithelial biomarker signature for idiopathic pulmonary fibrosis: an analysis from the multicentre PROFILE cohort study | Lancet RM | Maher | https://doi.org/10.1016/s2213-2600(17)30430-7 |
| 2017 | Genetic variants associated with susceptibility to idiopathic pulmonary fibrosis in people of European ancestry: a genome-wide association study | Lancet RM | Allen | https://doi.org/10.1016/s2213-2600(17)30387-9 |
| 2017 | Host–Microbial Interactions in Idiopathic Pulmonary Fibrosis | AJRCCM | Molyneaux | https://doi.org/10.1164%2Frccm.201607-1408OC |
| 2017 | The histone deacetylase inhibitor, romidepsin, as a potential treatment for pulmonary fibrosis | Oncotarget | Conforti | https://doi.org/10.18632/oncotarget.17114 |
| 2019 | Biomarkers of collagen synthesis predict progression in the PROFILE idiopathic pulmonary fibrosis cohort | Respir Res | Organ | https://doi.org/10.1186/s12931-019-1118-7 |
| 2019 | Patient-reported distress can aid clinical decision-making in idiopathic pulmonary fibrosis: analysis of the PROFILE cohort | ERJ | Stewart | https://doi.org/10.1183/13993003.01925-2018 |
| 2019 | Resequencing Study Confirms That Host Defense and Cell Senescence Gene Variants Contribute to the Risk of Idiopathic Pulmonary Fibrosis | AJRCCM | Moore | https://doi.org/10.1164/rccm.201810-1891OC |
| 2020 | Genome-Wide Association Study of Susceptibility to Idiopathic Pulmonary Fibrosis | AJRCCM | Allen | https://doi.org/10.1164/rccm.201905-1017oc |
| 2021 | BAL Is Safe and Well Tolerated in Individuals with Idiopathic Pulmonary Fibrosis: An Analysis of the PROFILE Study | AJRCCM | Molyneaux | https://doi.org/10.1164/rccm.202004-1138le |
| 2021 | Identification of a missense variant in SPDL1 associated with idiopathic pulmonary fibrosis | Commun Biol | Dhindsa | https://doi.org/10.1038%2Fs42003-021-01910-y |
| 2021 | Circulating fibrocytes are not disease-specific prognosticators in idiopathic pulmonary fibrosis | ERJ | Stewart | https://doi.org/10.1183/13993003.00172-2021 |
| 2022 | Biomarker signatures for progressive idiopathic pulmonary fibrosis | ERJ | Clynick | https://doi.org/10.1183/13993003.01181-2021 |
| 2022 | CYFRA 21-1 Predicts Progression in Idiopathic Pulmonary Fibrosis: A Prospective Longitudinal Analysis of the PROFILE Cohort | AJRCCM | Molyneaux | https://doi.org/10.1164/rccm.202107-1769oc |
| 2022 | Forced vital capacity trajectories in patients with idiopathic pulmonary fibrosis: a secondary analysis of a multicentre, prospective, observational cohort | Lancet DH | Fainberg | https://doi.org/10.1016/s2589-7500(22)00173-x |
| 2022 | A lung targeted miR-29 mimic as a therapy for pulmonary fibrosis | EBioMed | Chioccioli | https://doi.org/10.1016/j.ebiom.2022.104304 |
| 2022 | MUC5B rs35705950 minor allele associates with older age and better survival in idiopathic pulmonary fibrosis | Respirology | van der Vis | https://doi.org/10.1111/resp.14440 |
| 2023 | The Burden and Impact of Cough in Patients with Idiopathic Pulmonary Fibrosis: An Analysis of the Prospective Observational PROFILE Study | Annals ATS | Saunders | https://doi.org/10.1513/annalsats.202302-174oc |
| 2023 | Longitudinal lung function and gas transfer in individuals with idiopathic pulmonary fibrosis: a genome-wide association study | Lancet RM | Allen | https://doi.org/10.1016/s2213-2600(22)00251-x |
| 2023 | PCSK6 and Survival in Idiopathic Pulmonary Fibrosis | AJRCCM | Oldham | https://doi.org/10.1164%2Frccm.202205-0845OC |
| 2023 | Idiopathic Pulmonary Fibrosis Is Associated with Common Genetic Variants and Limited Rare Variants | AJRCCM | Peljto | https://doi.org/10.1164/rccm.202207-1331oc |
| 2024 | Deep Learning-based Segmentation of CT Scans Predicts Disease Progression and Mortality in IPF | AJRCCM | Thillai | https://doi.org/10.1164/rccm.202311-2185oc |
| 2024 | Association study of human leukocyte antigen variants and idiopathic pulmonary fibrosis | ERJ Open Res | Guillen-Guio | https://doi.org/10.1183%2F23120541.00553-2023 |
| 2024 | Cough Severity Visual Analogue Scale Assesses Cough Burden and Predicts Survival in Idiopathic Pulmonary Fibrosis | AJRCCM | Wu | https://doi.org/10.1164/rccm.202311-2169le |
| 2025 | The basement membrane repair response biomarker PRO-C4 predicts progression in idiopathic pulmonary fibrosis: analysis of the PFBIO and PROFILE cohorts | Thorax | Sand | doi: 10.1136/thorax-2024-221868 |
| 2025 | Rare variants and survival of patients with idiopathic pulmonary fibrosis: analysis of a multicentre, observational cohort study with independent validation | Lancet RM | Alonso-Gonzalez | https://doi.org/10.1016/S2213-2600(25)00045-1 |
