# Supplementary material for: Prospective study of fibrosis in the lung endpoints (PROFILE): characteristics of an incident cohort of patients with idiopathic pulmonary fibrosis
Source: BMJ Open Respir Res. 2026 Jan 28;13(1):e003763. doi: 10.1136/bmjresp-2025-003763 (PMC12853555; doi:10.1136/bmjresp-2025-003763)
Supplement: online supplemental file 2 [file bmjresp-13-1-s002.docx]

**Supplementary Materials**

**Supplemental Table 1: PROFILE study objectives and endpoints**

|  | **Objectives** | **Endpoints** |
| --- | --- | --- |
| **Primary** | To prospectively evaluate longitudinal disease behaviour in patients with IPF | FVC, FEV1, DLco, St George’s respiratory questionnaire, Leicester cough score, Six-minute walk test, concomitant medication |
| **Secondary** | To develop composite clinical endpoints for subsequent use in intervention studies in patients with IPF | CPI, GAP |
|  | To prospectively validate a panel of previously published biomarkers in patients with well characterized idiopathic fibrosing lung disease | Published biomarkers to be studied may include but are not limited to: CCL18, surfactants, metalloproteinases, TGFb-related markers. |
|  | To discover and qualify novel biomarkers and gene expression profiles for use in subsequent intervention studies in patients with IPF | Serum and plasma soluble protein multiplex platforms, blood transcriptomics, circulating microRNA |
|  | To compare circulating biomarkers to healthy age and sex-matched controls. | As for the IPF patients |
|  | To investigate genetic associations and epigenetic modifications which affect disease severity and progression | Blood DNA sample for genetic investigations |
|  | To investigate survival in IPF | NHS registry mortality data for up to 10 years |
| **Exploratory** | To prospectively assess the role of handheld spirometry in detecting early decline in lung function. | FVC |
|  | To discover and qualify novel biomarkers from bronchoalveolar lavage cells and fluid | Differential cell count, soluble markers in fluid, cell signalling |
|  | To quantify circulating fibrocytes at baseline and after 6 months follow-up | Number of fibrocytes in blood |

**Supplemental Table 2. PROFILE Design**

|  | screening | baseline | month 1 | month 3 | month 6 | month 12 | month 24 | month 36 |
| --- | --- | --- | --- | --- | --- | --- | --- | --- |
| Informed consent |  |  |  |  |  |  |  |  |
| Demographics |  |  |  |  |  |  |  |  |
| Medication |  |  |  |  |  |  |  |  |
| Lung function tests |  |  |  |  |  |  |  |  |
| Patient reported measures |  |  |  |  |  |  |  |  |
| 6MWT |  |  |  |  |  |  |  |  |
| Serum |  |  |  |  |  |  |  |  |
| Plasma |  |  |  |  |  |  |  |  |
| Blood for DNA |  |  |  |  |  |  |  |  |
| Blood for RNA |  |  |  |  |  |  |  |  |

**Supplemental Table 3. Effect allele frequency in cohort (N=534)**

| Chr | Position | rsID number | Gene | IPF risk allele | Alternate allele | RAF |
| --- | --- | --- | --- | --- | --- | --- |
| 1 | 150579566 | rs16837903 | *MCL1* | G | A | 0.886 |
| 1 | 155179278 | rs9426886 | *TRIM46* | T | A | 0.450 |
| 1 | 214482547 | rs12096551 | *PTPN14* | T | C | 0.812 |
| 3 | 14270502 | rs112271207 | *LSM3* | T | C | 0.076 |
| 3 | 44804157 | rs74341405 | *KIF15* | C | T | 0.054 |
| 3 | 169769713 | rs9811216 | *TERC* | C | T | 0.322 |
| 4 | 88892133 | rs6815970 | *FAM13A* | T | C | 0.281 |
| 5 | 1286401 | rs2736100 | *TERT* | A | C | 0.578 |
| 5 | 169588475 | rs116483731 | *SPDL1* | A | G | 0.023 |
| 6 | 7562999 | rs2076295 | *DSP* | G | T | 0.548 |
| 6 | 43386693 | rs1214761 | 6p21.2 | G | A | 0.519 |
| 6 | 35549503 | rs9348978 | *FKBP5* | G | A | 0.722 |
| 7 | 1936920 | rs2280550 | *MAD1L1* | G | A | 0.657 |
| 7 | 100020229 | rs35228488 | 7q22 | C | G | 0.437 |
| 7 | 129095384 | rs34288126 | *TPI1P2* | A | G | 0.142 |
| 8 | 119927966 | rs10808505 | *DEPTOR* | T | G | 0.598 |
| 9 | 106721382 | rs1388233 | 9q31.2 | A | G | 0.576 |
| 10 | 103882177 | chr10:103882177 | *STN1* | GCAAGTGA | G | 0.557 |
| 11 | 1219991 | rs35705950 | *MUC5B* | T | G | 0.342 |
| 12 | 95866696 | rs7957346 | *SNRPF* | A | C | 0.624 |
| 13 | 112886111 | rs3742238 | *ATP11A* | C | T | 0.825 |
| 15 | 40424054 | rs2304645 | *IVD* | C | G | 0.576 |
| 15 | 40621642 | rs2412541 | *KNL1* | G | T | 0.183 |
| 15 | 85744679 | rs11073517 | *AKAP13* | T | C | 0.340 |
| 16 | 34036 | rs367849850 | *IL9RP3* | G | GGGGAGCCTGGAAGCACAC | 0.069 |
| 17 | 45949182 | rs242561 | 17q21.31 | C | T | 0.830 |
| 19 | 4717660 | rs12610495 | *DPP9* | G | A | 0.338 |
| 19 | 5840608 | rs708686 | *FUT6* | T | C | 0.300 |
| 20 | 63652817 | rs112087793 | *STMN3* | C | T | 0.933 |
| 20 | 63694480 | rs115610405 | *RTEL1* | A | C | 0.033 |

Chr=chromosome, position given on build 38, risk allele=allele associated with increased risk of IPF, RAF=risk allele frequency. RAF not restricted to European ancestry.

**Supplemental Table 4. Unadjusted and adjusted mortality estimates**

| Risk factor |  | HR | 95%CI | p | aHR | adj95%CI | p |
| --- | --- | --- | --- | --- | --- | --- | --- |
| GAP | Stage I | ref |  |  | ref |  |  |
|  | Stage II | 2.73 | 2.11 to 3.53 | <0.001 | 1.91 | 1.40 to 2.62 | <0.001 |
|  | Stage III | 4.76 | 3.55 to 6.36 | <0.001 | 2.84 | 1.91 to 4.22 | <0.001 |
| Baseline ppFVC | Continuous % | 0.97 | 0.96 to 0.98 | <0.001 | 0.97 | 0.96 to 0.98 | <0.001 |
| Baseline ppDL_CO_ | Continuous % | 0.94 | 0.93 to 0.95 | <0.001 | 0.94 | 0.94 to 0.95 | <0.001 |
| Baseline age | Continuous year | 1.02 | 1.01 to 1.03 | 0.001 | 1.02 | 1.01 to 1.04 | <0.001 |
| Baseline CPI | Continuous index | 1.08 | 1.06 to 1.09 | <0.001 | 1.09 | 1.07 to 1.11 | <0.001 |
| Sex | Female | ref |  |  | ref |  |  |
|  | Male | 1.39 | 1.08 to 1.80 | 0.011 | 1.14 | 0.88 to 1.48 | 0.310 |
| 6MW desaturation | Normal saturation | ref |  |  | ref |  |  |
|  | Desaturation <88% | 2.70 | 2.06 to 3.55 | <0.001 | 1.96 | 1.47 to 2.61 | <0.001 |
| WHO | Normal | ref |  |  | ref |  |  |
|  | Underweight | 2.51 | 1.09 to 5.76 | 0.030 | 1.95 | 0.78 to 4.87 | 0.153 |
|  | Pre-obese | 0.96 | 0.73 to 1.25 | 0.752 | 1.05 | 0.80 to 1.38 | 0.731 |
|  | Obesity class I-III | 0.83 | 0.63 to 1.11 | 0.214 | 1.00 | 0.75 to 1.34 | 0.991 |
| Comorbidities | Not diagnosed | ref |  |  | ref |  |  |
|  | Hypertension | 1.28 | 1.04 to 1.57 | 0.020 | 1.05 | 0.84 to 1.31 | 0.660 |
|  | Diabetes | 1.07 | 0.81 to 1.40 | 0.646 | 0.97 | 0.73 to 1.28 | 0.811 |
|  | Heart disease | 1.13 | 0.86 to 1.47 | 0.393 | 1.14 | 0.86 to 1.50 | 0.367 |
|  | Cancer | 1.00 | 0.65 to 1.54 | 0.998 | 0.92 | 0.60 to 1.42 | 0.707 |
|  | Depression/anxiety | 1.00 | 0.72 to 1.39 | 0.993 | 1.31 | 0.93 to 1.84 | 0.125 |
|  | Any | 1.20 | 0.97 to 1.48 | 0.089 | 1.09 | 0.88 to 1.36 | 0.430 |
| Common variants |  |  |  |  |  |  |  |
|  | *MUC5B* rs35705950 | 0.84 | 0.70 to 1.02 | 0.073 | 0.68 | 0.55 to 0.83 | <0.001 |
|  | *TERT* rs2736100 | 1.09 | 0.92 to 1.29 | 0.543 | 1.03 | 0.87 to 1.23 | 0.707 |
|  | *TERC* rs9811216 | 1.04 | 0.88 to 1.23 | 0.663 | 1.07 | 0.90 to 1.26 | 0.472 |
|  | *RTEL1* rs115610405 | 1.05 | 0.69 to 1.61 | 0.817 | 0.94 | 0.61 to 1.45 | 0.791 |
| Telomere length | Q5 (mean 1.13, SD 0.17) | ref |  |  | ref |  |  |
|  | Q4 (mean 0.88 SD 0.04) | 1.42 | 0.99 to 2.04 | 0.056 | 1.27 | 0.88 to 1.83 | 0.206 |
|  | Q3 (mean 0.77 SD 0.03) | 1.49 | 1.03 to 2.13 | 0.032 | 1.32 | 0.91 to 1.92 | 0.142 |
|  | Q2 (mean 0.69 SD 0.02) | 1.38 | 0.96 to 1.99 | 0.083 | 1.20 | 0.83 to 1.75 | 0.333 |
|  | Q1 (mean 0.58 SD 0.06) | 1.69 | 1.18 to 2.40 | 0.004 | 1.72 | 1.20 to 2.48 | 0.003 |
| 12m FVC decline | <5% | ref |  |  | ref |  |  |
|  | >=5%<10% | 1.33 | 0.99 to 1.80 | 0.062 | 1.73 | 1.27 to 2.36 | <0.001 |
|  | >=10%<15% | 1.52 | 1.07 to 2.15 | 0.019 | 1.86 | 1.30 to 2.67 | 0.001 |
|  | >15% | 2.90 | 1.08 to 4.06 | <0.001 | 3.54 | 2.52 to 4.99 | <0.001 |
| 12m ppDL_CO_ decline | <5% | ref |  |  | ref |  |  |
|  | >=5%<10% | 1.10 | 0.77 to 1.58 | 0.607 | 1.03 | 0.72 to 1.48 | 0.857 |
|  | >=10%<15% | 1.51 | 1.04 to 2.20 | 0.029 | 1.54 | 1.06 to 2.24 | 0.025 |
|  | >15% | 2.87 | 2.01 to 4.09 | <0.001 | 2.67 | 1.87 to 3.82 | <0.001 |

Unadjusted hazard ratio (HR) and adjusted hazard ratio (aHR) presented with 95% confidence intervals from cox proportional hazard models censored at five years; aHR models adjusted for baseline percent predicted forced vital capacity (ppFVC), age and sex. IPF common variant associations were restricted to participants with gene array data and genetically inferred European Ancestry, further adjusted for first three genetic principal components, with risk allele included in an additive model. Telomere length presented as quintiles (Q) with mean length and standard deviation (SD). Gender-Age-Physiology Index (GAP); percent predicted carbon monoxide diffusion capacity (ppDL_CO_); Six-minute walk test (6MWT), SpO2 desaturation <88%. WHO nutritional status underweight class n=7. Hazards for 12-month relative decline categories of FVC and ppDL_CO_ based on <5%, 5% to <10%, 10% to <15%, ≥15% categories estimated using mixed effect models, adjusted for baseline age, sex and ppFVC, specified with restricted cubic splines using three knots, a random intercept for participant and random effect for study visit.

**Supplemental Figure 1. PROFILE comorbidities (N=580)**

Percent of participants with major comorbidities

**Supplemental Figure 2: PROFILE baseline concomitant medication (N=580)**

Percent of cohort with concomitant medication records.

**Supplemental Figure 3. Unadjusted overall survival (N=632)**

Kaplan-Meier plot of survival in the overall PROFILE cohort.

**Supplemental Figure 4**

**
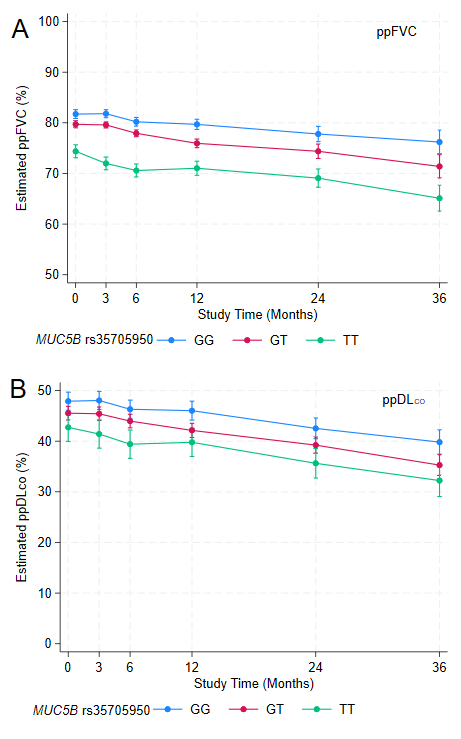
**

Lung function change by *MUC5B* genotype estimated with mixed effect model in participants reporting white ethnicity, adjusted for first three genetic principal components, baseline age, sex and baseline ppFVC, specified in additive model with restricted cubic splines using three knots, a random intercept for participant and random effect for study visit. A) Longitudinal percent predicted forced vital capacity (ppFVC). B) Longitudinal percent predicted diffusion capacity of carbon monoxide (ppDL_CO_).

**Supplemental Figure 5**

**
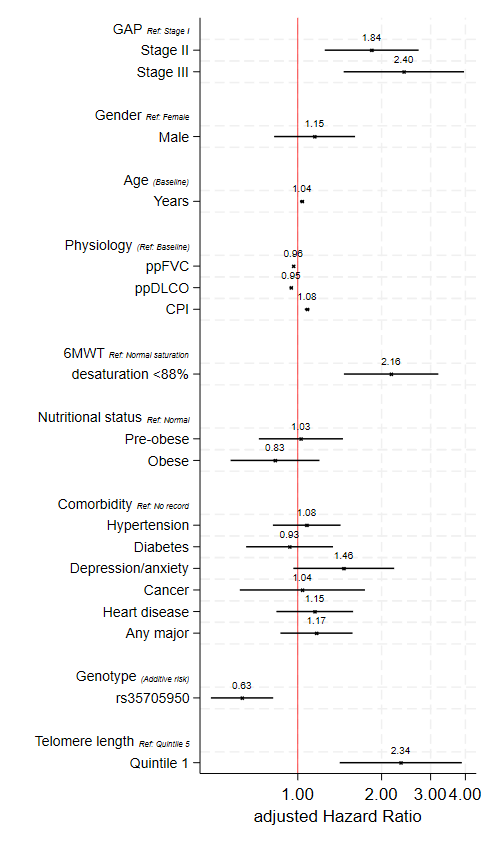
**

Sensitivity analysis performed in 400 participants with no evidence of anti-fibrotic use, or steroid/immunosuppressive use. Estimates modelled using cox proportional hazards adjusted for continuous age at baseline, sex and continuous baseline percent predicted forced vital capacity (ppFVC). Follow-up time censored at five years. MUC5B rs35705950 was restricted to participants reporting European ancestry further adjusted for first 3 genetic principal components, with risk allele included in an additive model. Telomere length presented as quintiles (Q) with Q5 representing greatest length and Q1 representing shortest length. Gender-Age-Physiology Index (GAP); percent predicted carbon monoxide diffusion capacity (ppDLCO); Six-minute walk test (6MWT), SpO2 desaturation <88%.

**Supplemental Figure 6**

**
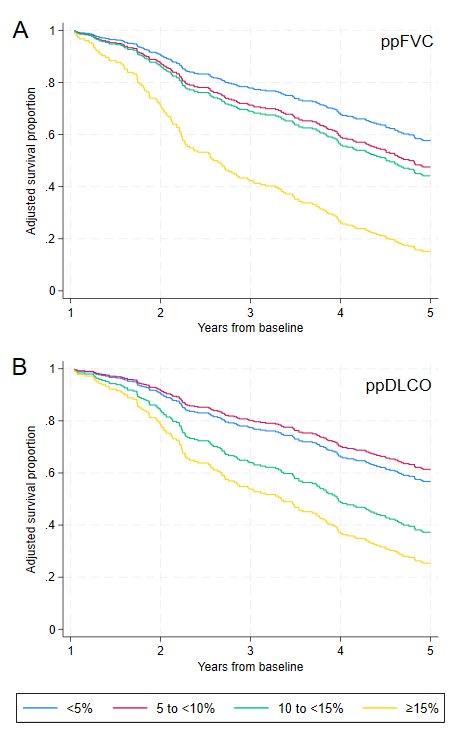
**

Sensitivity analysis performed in individuals with no evidence of antifibrotic use or steroid/immunosuppressive use. Adjusted survival curves for 12-month relative decline categories of A) percent predicted forced vital capacity (ppFVC); B) percent predicted carbon monoxide diffusion capacity (ppDLCO); based on <5% (blue), 5% to <10% (red), 10% to <15% (green), ≥15% (yellow) categories estimated using mixed effect models, adjusted for baseline age, sex and ppFVC, specified with restricted cubic splines using three knots, a random intercept for participant and random effect for study visit.
